# Supplementary material for: Natural history of nonhuman primates after conjunctival exposure to Ebola virus
Source: Sci Rep. 2023 Mar 13;13:4175. doi: 10.1038/s41598-023-31027-7 (PMC10011569; doi:10.1038/s41598-023-31027-7)
Supplement: Supplementary file 1 — Supplementary Information. [file 41598_2023_31027_MOESM1_ESM.docx]

*
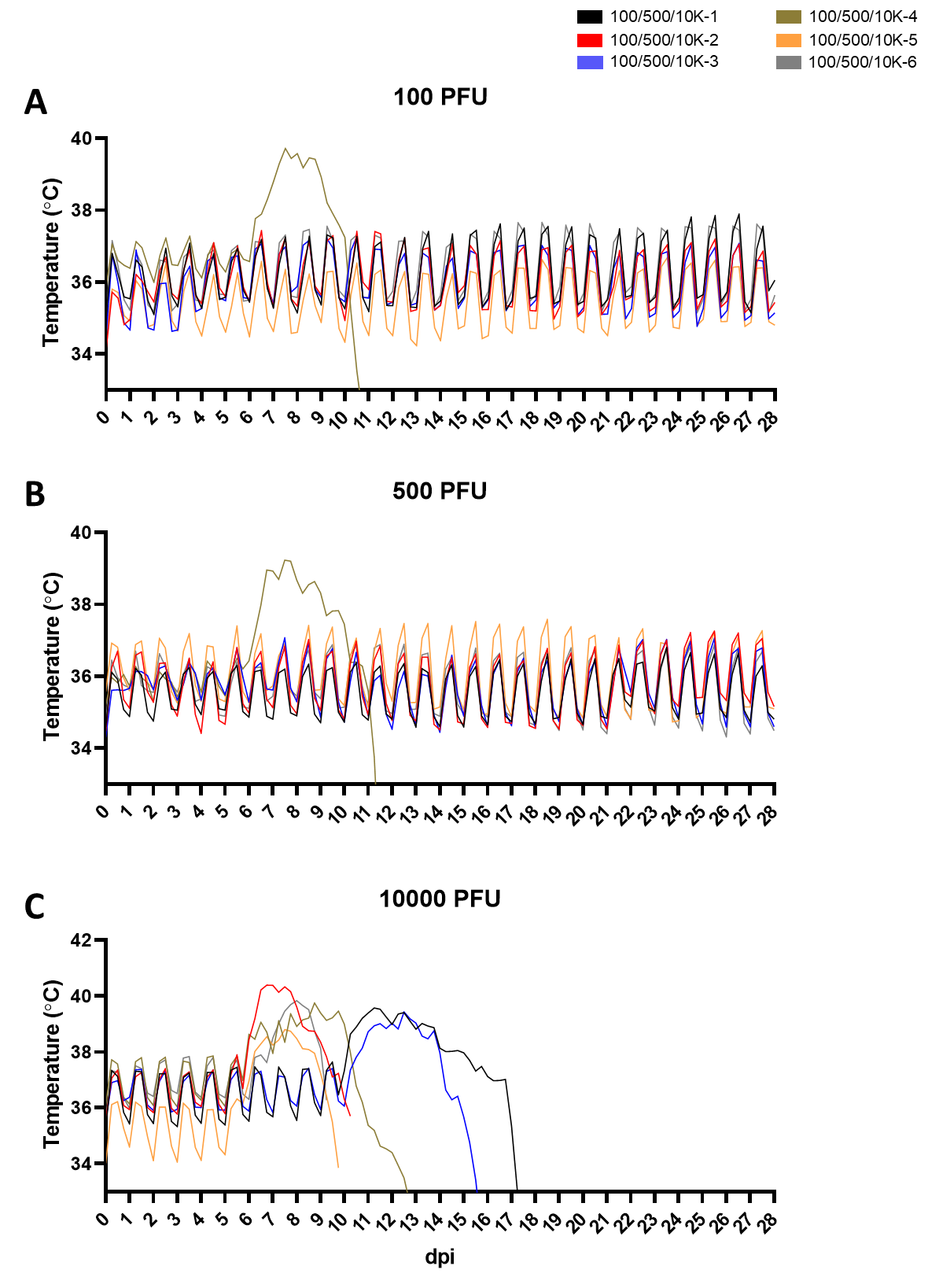
*

**Fig S1:** **Core body temperature telemetry of EBOV-challenged cynomolgus macaques.** Core body temperatures from each macaque were measured in 15-minute increments throughout the study duration via intraperitoneal implantation of telemetric temperature loggers (described in “Methods”). Each data point represents the 6-hour rolling average (i.e., average of 24 measurements within a 6 hour block) for each animal. **(A)** Core temperature measurements for the 100 PFU challenge cohort; **(B)** Core temperature measurements for the 500 PFU challenge cohort; **(C)** Core temperature measurements for the 10,000 PFU challenge cohort.


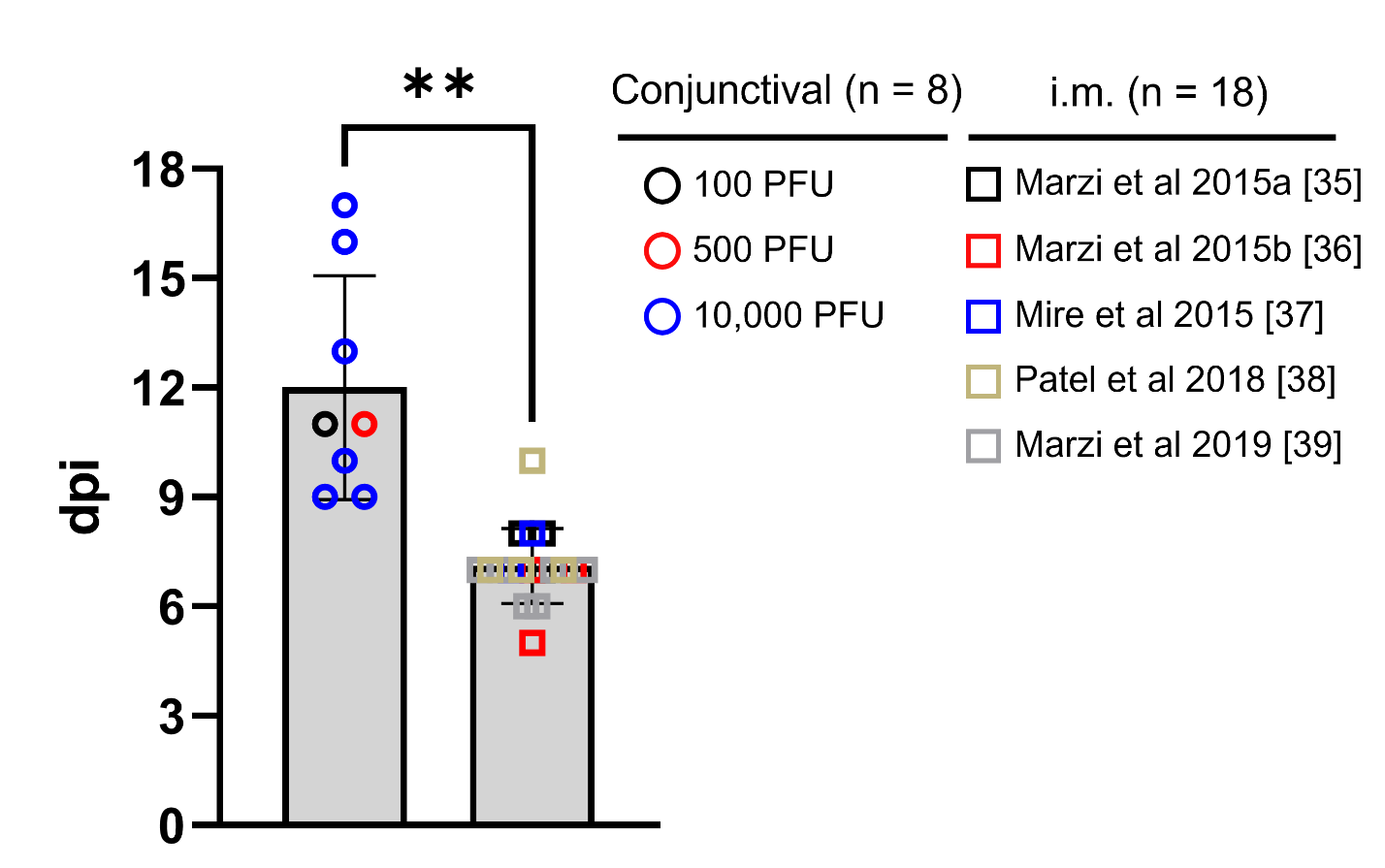


**Figure S2: Comparison of survival time between cynomolgus macaques challenged with EBOV Makona via conjunctival vs. i.m. routes.** The mean time to death (MTD) of animals which succumbed following conjunctival challenge was compared to that of positive control cynomolgus macaques from the published literature challenged with the C07 isolate of EBOV Makona via the i.m. route. Statistical comparison was performed using Welch’s t-test. **: p = 0.003. Bracketed numbers following cited literature refer to main paper in-text citations. dpi = days post-infection.

*
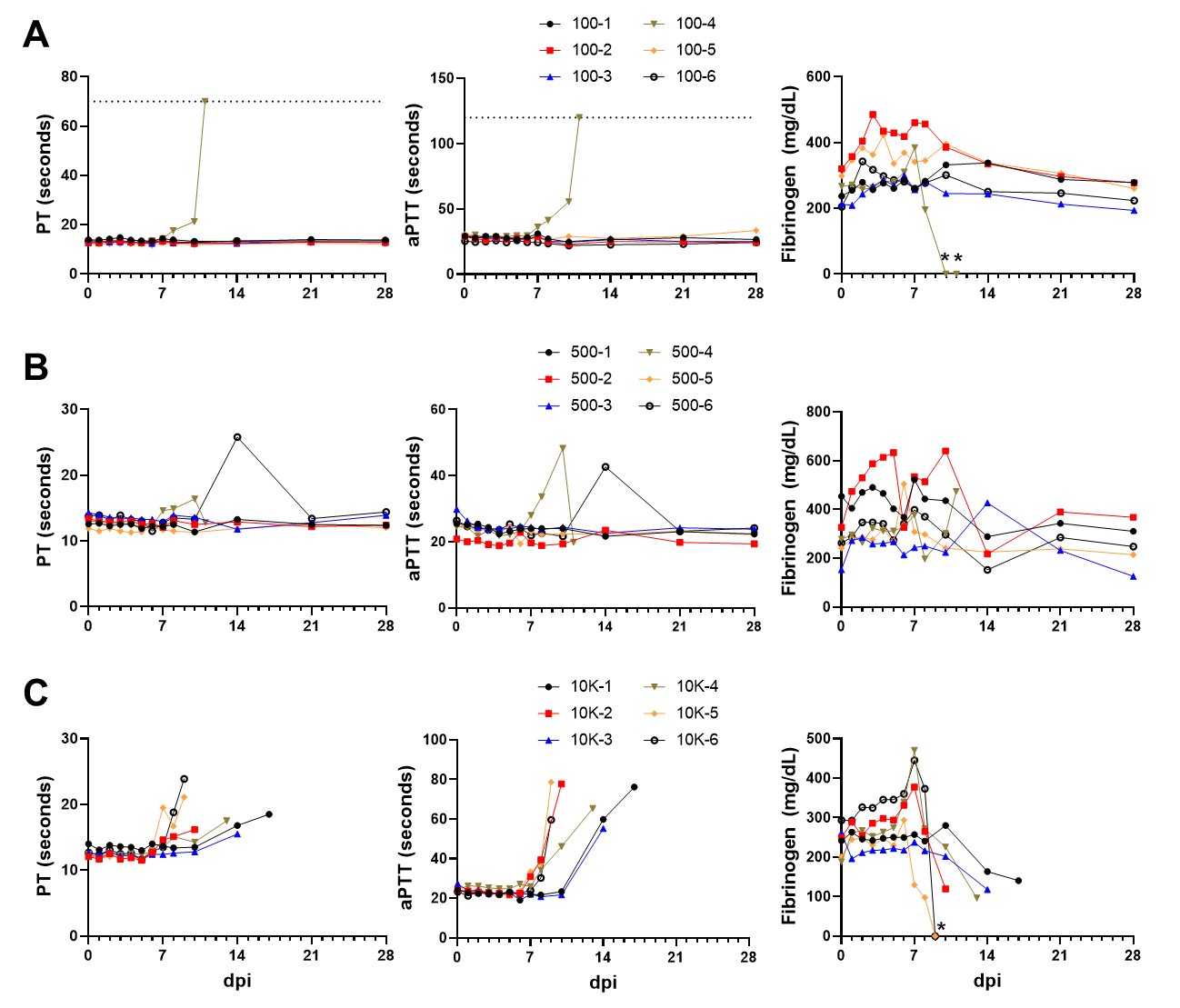
*

**Figure S3: Coagulopathy profiles of cynomolgus macaques challenged with EBOV.** Measurement of coagulation parameters for cynomolgus macaques challenged with 100 PFU **(A)**, 500 PFU **(B)**, or 10,000 PFU **(C)** EBOV-Makona. For each animal, the prothrombin time (PT), activated partial thromboplastin time (aPTT), and circulating fibrinogen were measured at the indicated timepoints. Horizontal dashed lines in the PT and aPTT panels in **(A)** indicate the upper limit of detection for the assay. Asterisks in the fibrinogen panels in **(A)** and **(C)** indicate undetectable levels.

**
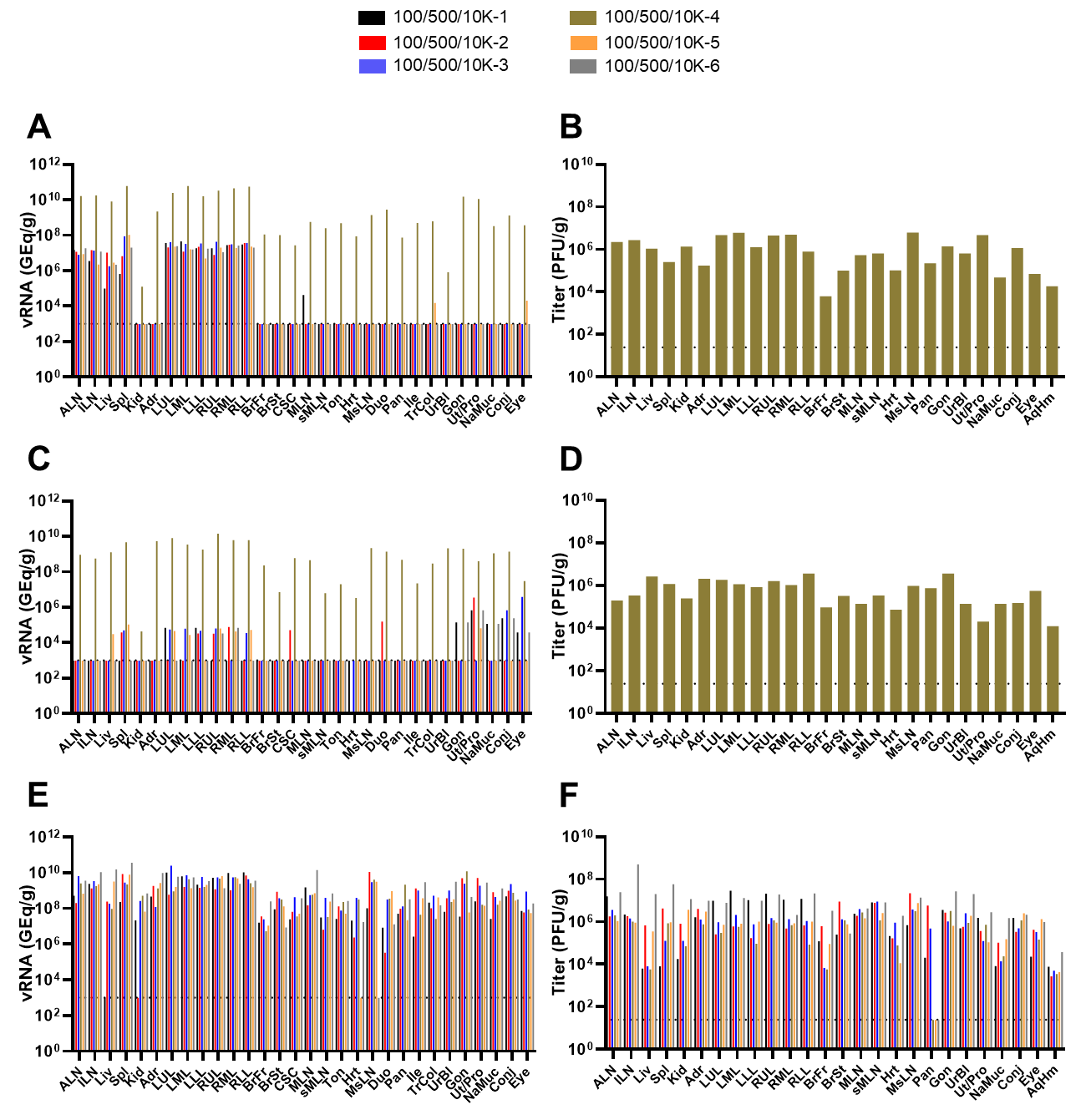
**

**Figure S4: Determination of tissue viral burden in EBOV-challenged macaques.** Viral load was determined by RT-qPCR detection of EBOV vRNA **(A,C,E)** and plaque titration (**B,D,E)** from selected tissues harvested at necropsy. For all panels, individual data points represent the mean of two technical replicates. Dashed horizontal lines indicate the limit of detection (LOD) for the assay (1000 GEq/g tissue for RT-qPCR; 25 PFU/g tissue for plaque titration). Values below the LOD for RT-qPCR and plaque assays were plotted as 999 GEq/g and 24 PFU/g tissue, respectively. Missing data indicates the tissue was not collected or assayed for that subject. Abbreviations for tissues: ALN: Axillary lymph node; ILN: inguinal lymph node; Liv: liver; Spl: spleen; Kid: kidney; Adr: adrenal gland; LUL: left upper lung; LML: left middle lung; LLL: left lower lung; RUL: right upper lung; RML: right middle lung; RLL: right lower lung; BrFr: brain frontal cortex; BrSt: brain stem; CSC: cervical spinal cord; MLN: mandibular lymph node; sMLN: submandibular lymph node; Ton: tonsil; Hrt: heart; MsLN: mesenteric lymph node; Duo: duodenum; Pan: pancreas; Ile: ileum; TrCol: transverse colon; UrBl: urinary bladder; Gon: gonad; Ut/Pro: uterus/prostate; NaMuc: nasal mucosa; Conj: conjunctiva.


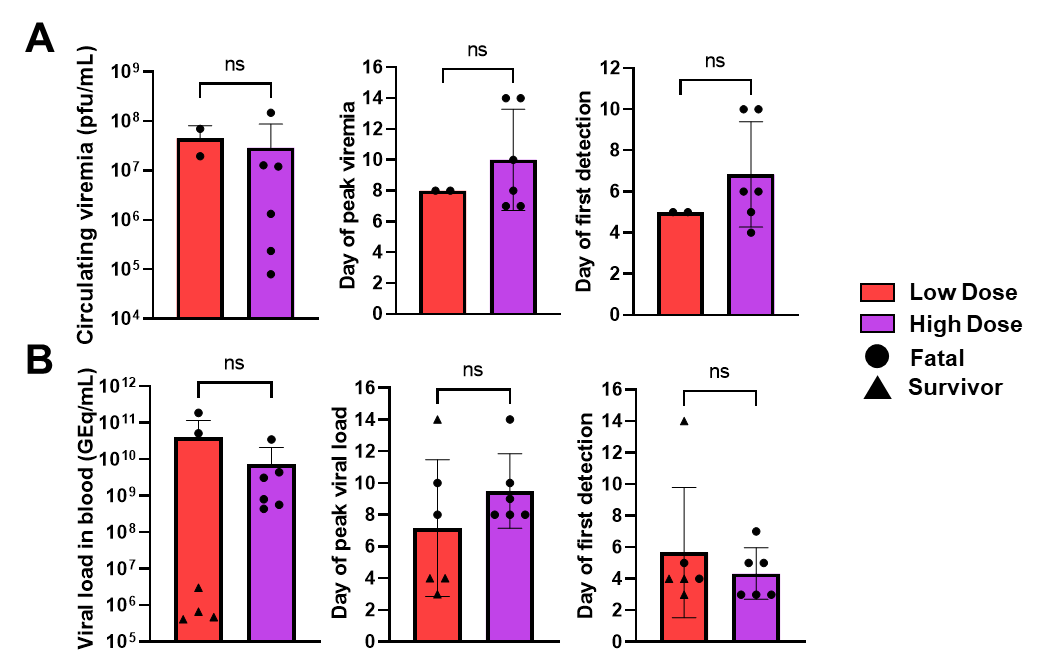


**Figure S5: Comparison of viral RNA and infectious virus in whole blood and plasma from EBOV-challenged cynomolgus macaques.** The peak viral load (irrespective of which day it was detected), the day peak viral load was detected, and the day infectious virus **(A)** or EBOV vRNA **(B)** was first detected in whole blood or plasma, respectively, was compared between macaques challenged with “low” (100 and 500 PFU) or “high” (10,000 PFU) doses of EBOV. Statistical significance was determined by unpaired t-test with Welch’s correction.


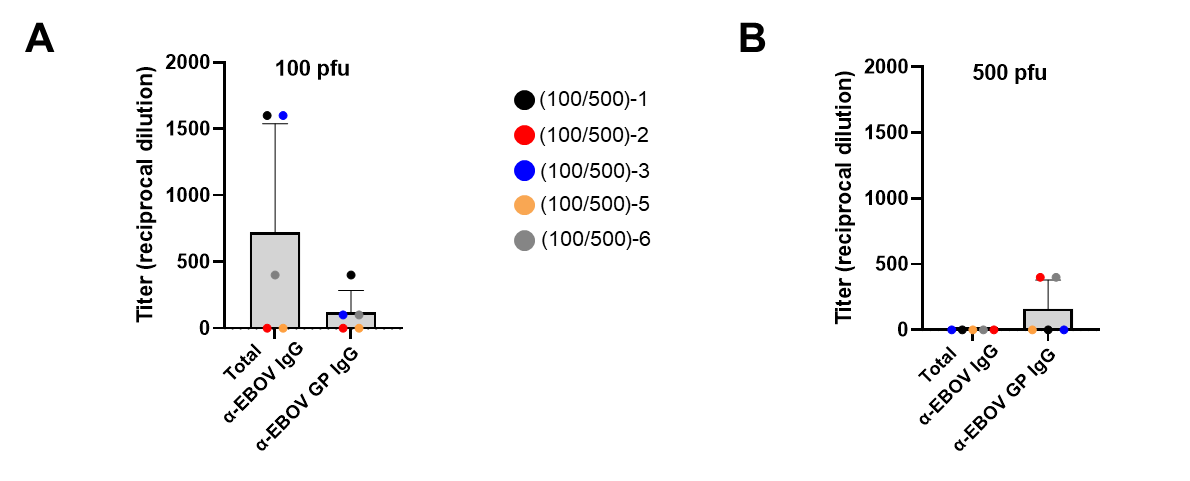


**Fig S6: Determination serum IgG titers in EBOV-challenged macaques.** ELISA-based quantification of serum anti-IgG titers from survivors at the study endpoint (28 dpi). IgG titers were measured against irradiated virus or GP antigen. Bars represent the mean IgG titer ± SD, values from individual animals are represented as colored circles within the bars.

| **Subject No.** | **Sex** | **Challenge dose (pfu; target/actual)** | **Clinical illness** | **Clinical pathology** |
| --- | --- | --- | --- | --- |
| **100-1** | M | 100/115 | Decreased appetite (d2-4,6,22); hypothermia (d14,21). Subject survived to study endpoint (d28). | Lymphocytopenia (d3); monocytopenia (d3,4); granulocytopenia (d6); > 2-fold ↑ in AST (d4). |
| **100-2** | M |  | None. Subject survived to study endpoint (d28). | Leukocytosis (d2); monocytosis (d2,3,5-8,10,28); granulocytosis (d1,2,6,7); granulocytopenia (d28); > 2-fold ↑ in ALT (d10); > 2-fold ↑ in CRP (d2,7). |
| **100-3** | F |  | Decreased appetite (d1-10). Subject survived to study endpoint (d28). | Granulocytopenia (d28); monocytosis (d1-3,5,7,10,28). |
| **100-4** | F |  | Decreased appetite (d0-2,7); fever (d7,8); anorexia (d8-11); petechial rash (d10,11); recumbency (d11); bradypnea (d11). Subject euthanized on d11. | Monocytosis (d0-6); granulocytopenia (d1-4); granulocytosis (d7,8); lymphocytopenia (d7,8,10); thrombocytopenia (d10,11); erythrocytopenia (d11); hypoglycemia (d11); > 2-fold ↑ in BUN (d10,11); > 2-fold ↑ in CRE (d11); hypoalbuminemia (d10,11); hypoproteinemia (d10,11); > 2-fold ↑ in ALT (d8,10,11); > 7-fold ↑ in AST (d8,10,11); > 6-fold ↑ in ALP (d10,11); > 4-fold ↑ in GGT (d10,11); hypoamylasemia (d8,10); > 4-fold ↑ in CRP (d4,6,7,8,10,11). |
| **100-5** | M |  | None. Subject survived to study endpoint (d28). | Monocytosis (d2,6,10,14); > 2-fold ↑ in CRP (d4). |
| **100-6** | F |  | Decreased appetite (d1-3). Subject survived to study endpoint (d28). | Granulocytopenia (d4,8,10,14,21,28); monocytosis (d2,4); > 2-fold ↑ in ALT (d4-8); > 2-fold ↑ in AST (d1-3). |
|  |  |  |  |  |
| **500-1** | M | 500/475 | Epistaxis (d3). Subject survived to study endpoint (d28). | Granulocytopenia (d0,2,4,7,10,14,21,28); thrombocytosis (d3,4,6-8,10,14,21); lymphocytosis (d0-8,10,21); monocytosis (d1,2,4,), monocytopenia (d28); hypoglycemia (d28). |
| **500-2** | M |  | Decreased appetite (d1,3,7-9). Subject survived to study endpoint (d28). | Monocytosis (d0); granulocytopenia (d6,14,21,28); lymphocytopenia (d28); > 2-fold ↑ in CRP (d3,4). |
| **500-3** | F |  | Decreased appetite (d2-4,8,9). Subject survived to study endpoint (d28). | Lymphocytopenia (d2,14,28); granulocytopenia (d14); monocytopenia (d1-3,6-8,10,21,28). |
| **500-4** | M |  | Decreased appetite (d7); anorexia (d8-11); petechial rash (d8-11); depression (d8-11); recumbency (d11). Subject euthanized on d11. | Monocytosis (d1,4-6); granulocytosis (d7,8); monocytopenia (d8,10,11); lymphocytopenia (d7,8,10,11); thrombocytopenia (d7,8,10,11); granulocytopenia (d11); anemia (d11); hypoglycemia (d11); > 5-fold ↑ in BUN (d10,11); > 5-fold ↑ in CRE (d10,11); hypocalcemia (d11); hypoalbuminemia (d10,11); hypoproteinemia (d11); ); > 5-fold ↑ in ALT (d10,11); > 4-fold ↑ in AST (d8); > 17-fold ↑ in AST (d10); > 29-fold ↑ in AST (d11); > 2-fold ↑ in ALP (d10); > 2-fold ↑ in GGT (d10,11); hypoamylasemia (d7,8); > 3-fold ↑ in CRP (d6,11); > 3-fold ↑ in CRP (d6,11); > 17-fold ↑ in CRP (d7,8,10). |
| **500-5** | F |  | Decreased appetite (d1-3,8,9). Subject survived to study endpoint (d28). | Granulocytopenia (d1,2,4-6,8,10,14,21); monocytopenia (d3,5-8,10,14,21,28); anemia (d8,10,14). |
| **500-6** | F |  | Decreased appetite (d1-5,7,8). Subject survived to study endpoint (d28). | Monocytosis (d1); granulocytosis (d1); granulocytopenia (d14,21,28); > 2-fold ↑ in CRP (d6). |
|  |  |  |  |  |
| **10K-1** | M | 10,000/9,625 | Decreased appetite (d11); anorexia (d12-17); petechial rash (d13-17); ataxia (d13-17); depression (d15-17); lethargy (d17); recumbency (d17); epistaxis (d17). Subject euthanized on d17. | Lymphocytopenia (d10,14,17); thrombocytopenia (d10,14,17); monocytopenia (d6,10,14); granulocytosis (d17); anemia (d10); >2-fold ↑ in BUN (d14,17); >4-fold ↑ in CRE (d14); >15-fold ↑ in CRE (d17); hypocalcemia (d14,17); hypoalbuminemia (d14,17); > 2-fold ↑ in ALT (d14,17); > 13-fold ↑ in AST (d14-17); > 5-fold ↑ in ALP (d14,17); > 2-fold ↑ in GGT (d14,17); > 3-fold ↑ in CRP (d10,17). |
| **10K-2** | F |  | Decreased appetite (d8); anorexia (d9,10); petechial rash (d8-10); depression (d9,10); hematochezia (d10); bleeding from venipuncture site (d10); hypothermia (d10). Subject euthanized on d10. | Leukocytosis (d7,10); granulocytosis (d6-8,10); thrombocytopenia (8,10); monocytopenia (d0-7); anemia (d8); >6-fold ↑ in BUN (d10); >6-fold ↑ in CRE (d10); hypocalcemia (d10); hypoalbuminemia (d10); >2-fold ↑ in ALT (d8,10); >8-fold ↑ in AST (d8,10); >2-fold ↑ in ALP (d10); >3-fold ↑ in GGT (d10); hypoamylasemia (d7,8,10); >15-fold ↑ in CRP (d7,8,10). |
| **10K-3** | F |  | Decreased appetite (d12,13); anorexia (d14,15); petechial rash (d14,15); depression (d15). Subject succumbed on d16. | Leukocytosis (d5,16); granulocytosis (d2-6,8,10,14,16); lymphocytopenia (d14); thrombocytopenia (d14); monocytopenia (d0,2,5,6,14); hypoglycemia (d16); >5-fold ↑ in BUN (d16); >7-fold ↑ in CRE (d16); hypoalbuminemia (d16); >7-fold ↑ in ALT (d16); >7-fold ↑ in AST (d14); >31-fold ↑ in AST (d16); >2-fold ↑ in ALP (d14,16); >2-fold ↑ in GGT (d14,16); hypoamylasemia (d14); >7-fold ↑ in CRP (d14,16). |
| **10K-4** | F |  | Fever (d6-8); decreased appetite (d8,10); anorexia (d9,11-13); depression (d12,13); petechial rash (d13); diarrhea (d13); lethargy (d13); recumbency (d13). Subject euthanized on d13. | Monocytosis (d1,4); thrombocytosis (0-8,10,13); lymphopenia (d8); lymphocytosis (d10); hyperglycemia (d6); >3-fold ↑ in BUN (d13); 2-fold ↑ in CRE (d10); hypocalcemia (d13); hypoalbuminemia (d10,13); hypoproteinemia (d13); >3-fold ↑ in AST (d8,10); >12-fold ↑ in AST (d13); >2-fold ↑ in GGT (d10,13); hypoamylasemia (d7,8,10,13); >2-fold ↑ in CRP (d6,10,13); >17-fold ↑ in CRP (d7,8). |
| **10K-5** | M |  | Decreased appetite (d0-6); anorexia (d7-9); petechial rash (d7-9); fever (d7,8); depression (d8,9); lethargy (d8,9); recumbency (d9). Subject euthanized on d9. | Monocytopenia (d3,5-9); lymphocytopenia (d6,7); thrombocytopenia (d7-9); leukocytosis (d9); lymphocytosis (d9); monocytosis (d9); granulocytosis (d9); > 6-fold ↑ in BUN (d9); > 2-fold ↑ in CRE (d8,9); hypoalbuminemia (d9); > 5-fold ↑ -in ALT (d9); > 5-fold ↑ in AST (d7-9); > 3-fold ↑ in ALP (d8,9); > 2-fold ↑ in GGT (d8,9); hypoamylasemia (d7,8); > 3-fold ↑ in CRP (d6-9). |
| **10K-6** | M |  | Decreased appetite (d0-7); fever (d7,8); anorexia (d8,9); petechial rash (d8,9); depression (d9); bradypnea (d9); recumbency (d9). Subject euthanized on d9. | Granulocytopenia (d0,1); monocytosis (d1,3); monocytopenia (d4,6,7); lymphocytopenia (d7,8); thrombocytopenia (d8,9); hypoglycemia (d9); > 4-fold ↑ in BUN (d9); > 5-fold ↑ in CRE (d9); hypocalcemia (d9); hypoalbuminemia (d9); > 20-fold ↑ -in ALT (d9); > 83-fold ↑ -in AST (d9); > 2-fold ↑ in ALP (d8,9); > 2-fold ↑ in GGT (d8,9); hypoamylasemia (d8); > 13-fold ↑ in CRP (d8,9). |
|  |  |  |  |  |

**Table S1. Clinical description and outcome of EBOV-challenged cynomolgus macaques**

Days after EBOV challenge are in parentheses. All parameters are reported in relation to baseline values (8 days prior to infection; d -8). Lymphopenia, granulopenia, monocytopenia, erythrocytopenia, and thrombocytopenia are defined by a ≥35% drop in numbers of lymphocytes, granulocytes, monocytes, and platelets from baseline, respectively. Leukocytosis, monocytosis and granulocytosis are defined by a two-fold or greater increase in numbers of white blood cells over base line. Thrombocytosis is defined by a two-fold or greater increase in numbers of platelets over baseline. Anemia is defined as a ≥35% decrease in red blood cells, hemoglobin, and hematocrit volume from baseline. Fever is defined as a temperature more than 2.5 °F over baseline, or at least 1.5 °F over baseline and ≥ 103.5 °F. Hypothermia is defined as a temperature ≤3.5°F below baseline. Hyperglycemia is defined as a two-fold or greater increase in levels of glucose. Hypoglycemia is defined by a ≥25% decrease in levels of glucose. Hypocalcemia is defined by a ≥25% decrease in levels of serum calcium. Hypoalbuminemia is defined by a ≥25% decrease in levels of albumin. Hypoproteinemia is defined by a ≥25% decrease in levels of total protein. Hypoamylasemia is defined by a ≥25% decrease in levels of serum amylase. (ALT) alanine aminotransferase, (AST) aspartate aminotransferase, (ALP) alkaline phosphatase, (CRE) Creatinine, (CRP) C-reactive protein, (Hct) hematocrit, (Hgb) hemoglobin
